# Supplementary figures and images for: Distribution and phylogeography of the genus Mattirolomyces with a focus on the Asian M. terfezioides haplotypes
Source: PeerJ. 2022 Jul 26;10:e13511. doi: 10.7717/peerj.13511 (PMC9336612; doi:10.7717/peerj.13511)

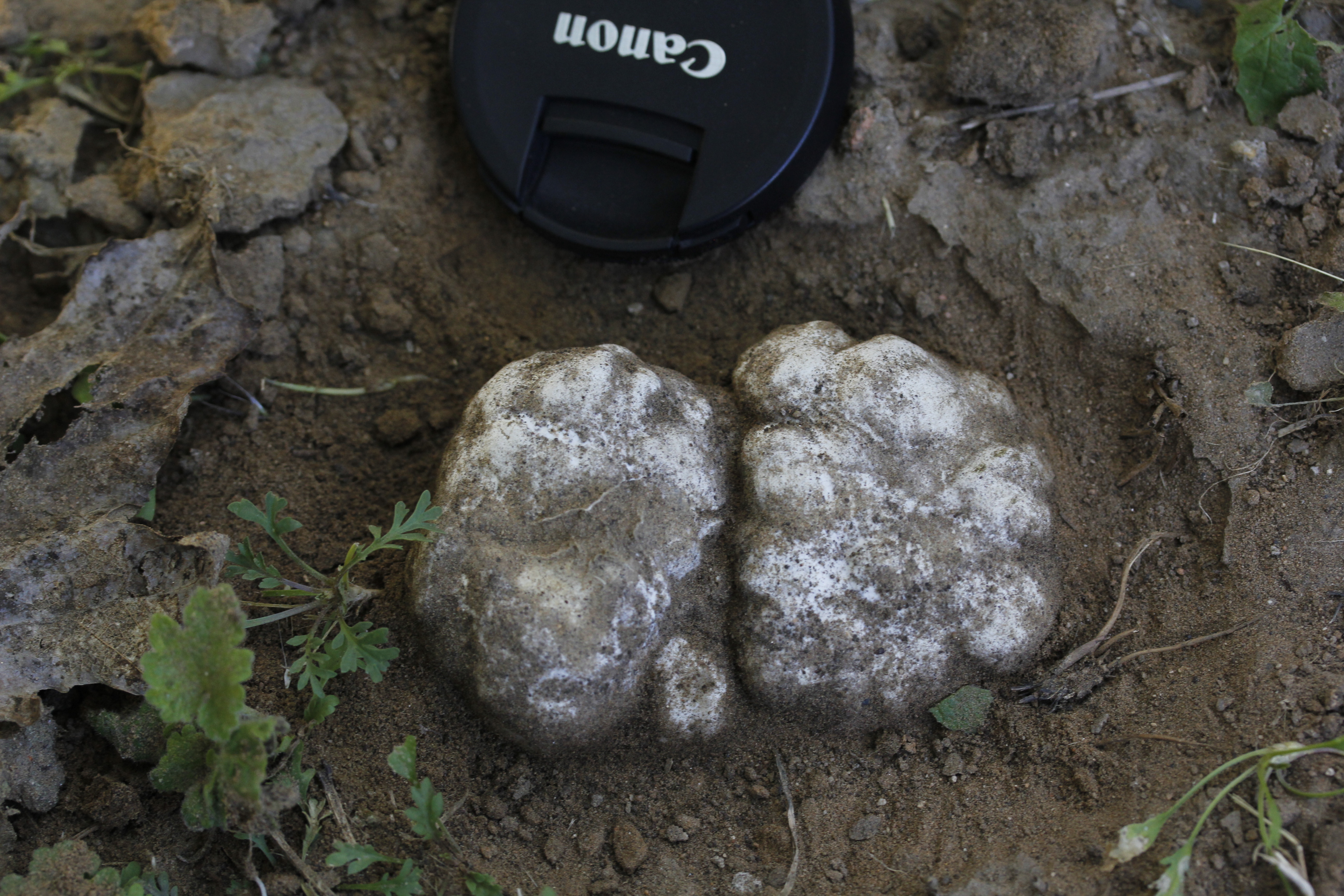

Supplement: Supplemental Information 1 — Photo taken at the collection site in the desert areas of Inner Mongolia, China. [file peerj-10-13511-s001.jpg]

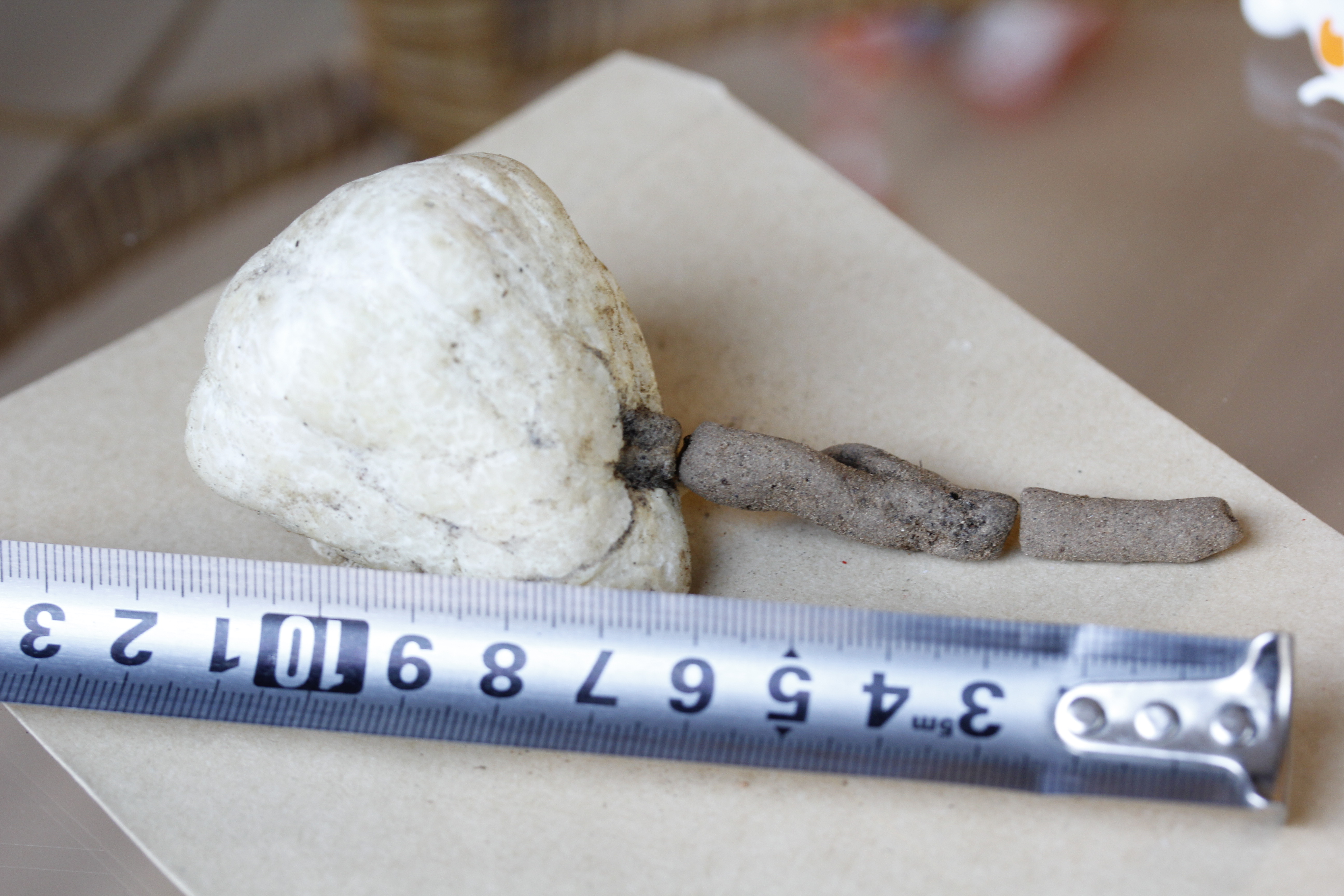

Supplement: Supplemental Information 2 [file peerj-10-13511-s002.jpg]

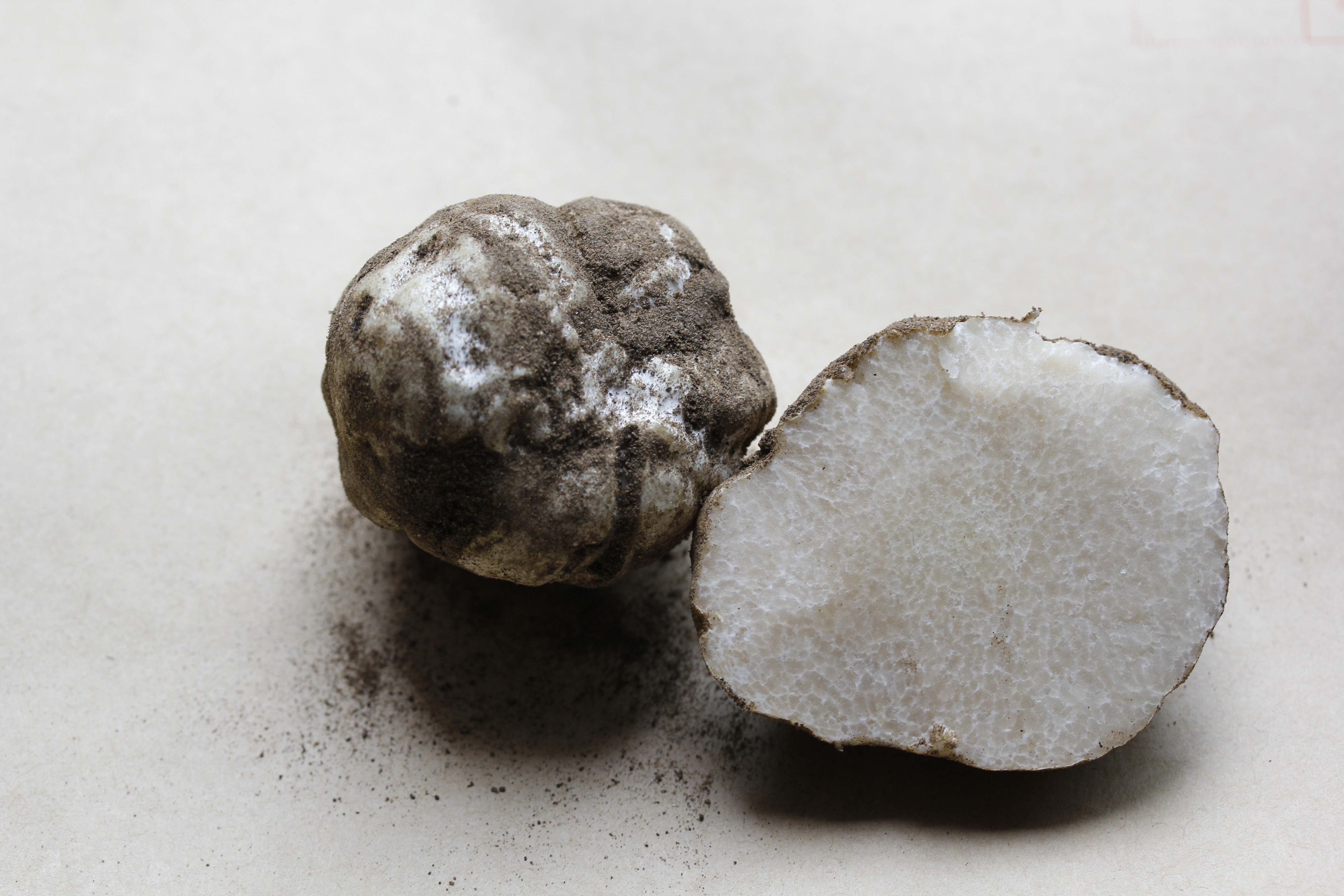

Supplement: Supplemental Information 3 — Cross section of an ascocarp. [file peerj-10-13511-s003.jpg]

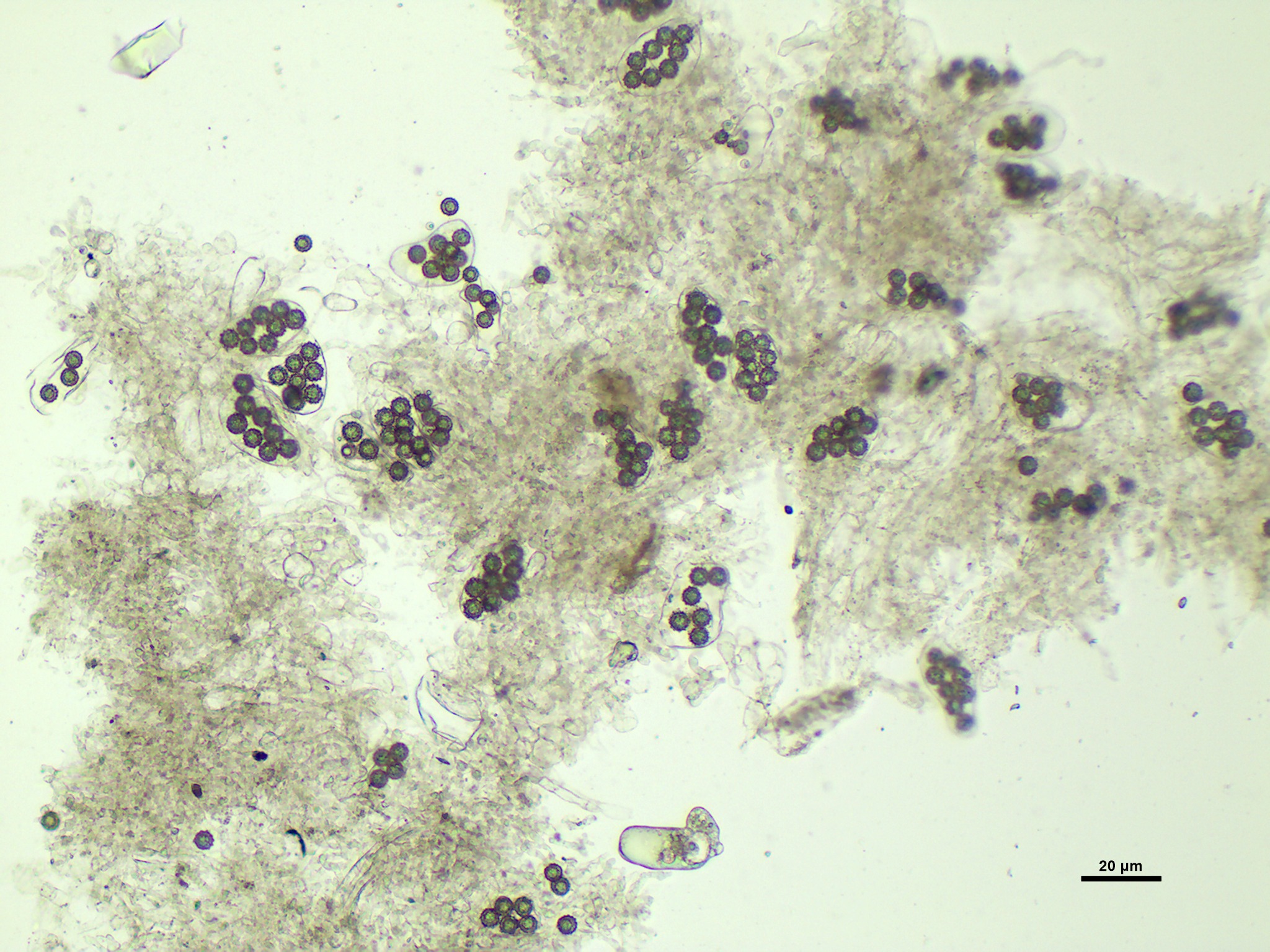

Supplement: Supplemental Information 4 — Microscopic slide made in Cotton blue. Asci opaque, containing 8-spores, irregularly shaped. Spores hyaline to pale yellow when native, in Cotton blue staining blue-gray, globose, with ornamentation. [file peerj-10-13511-s004.jpg]

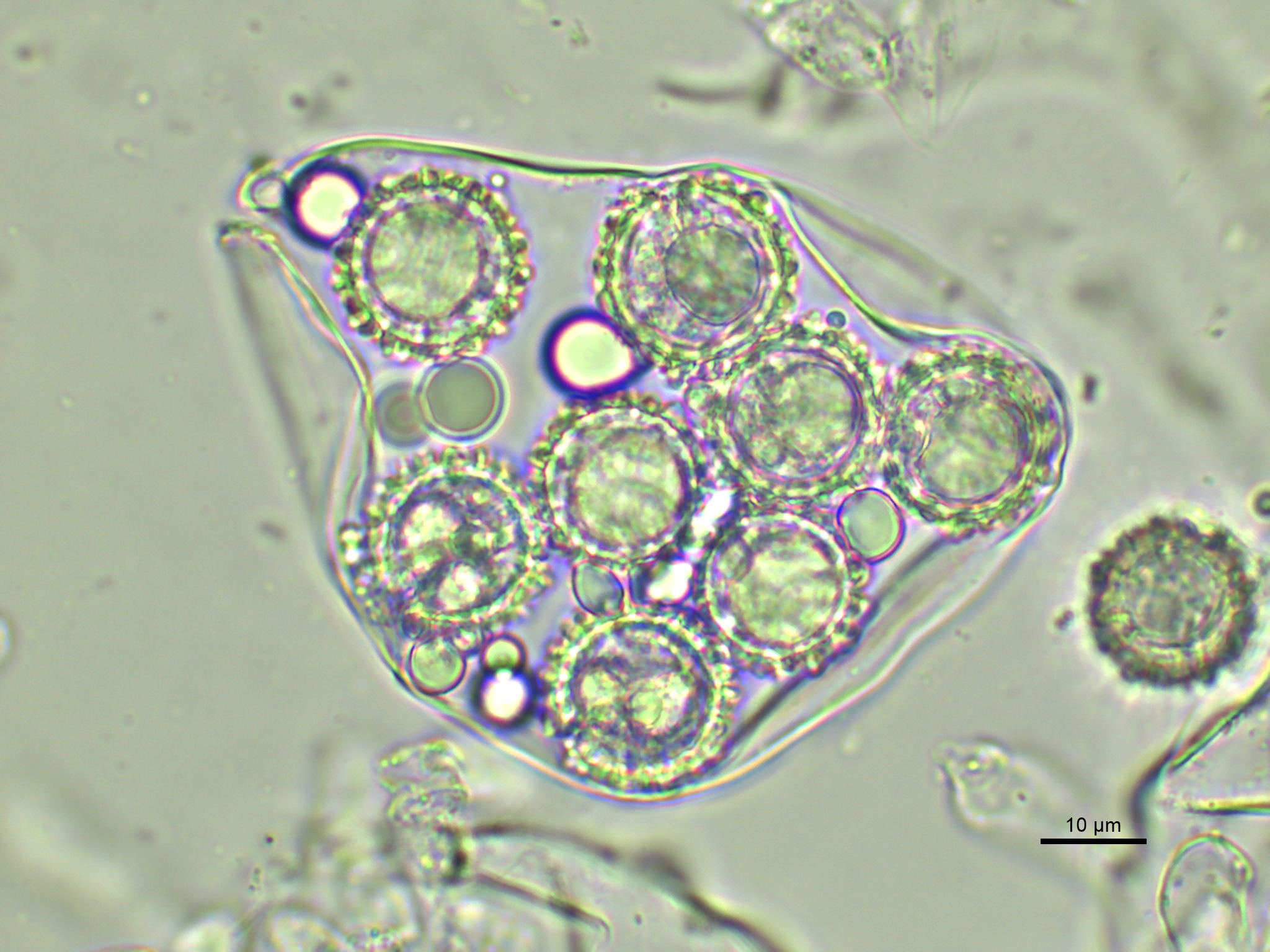

Supplement: Supplemental Information 5 — Microscopic slide made in water. Spores hyaline to pale yellow, globose, 14–19 (22) μm in diameter, ornamentation of spores ornamentation 1–4 μm high, forming blunt spines connected in an irregular alveolate reticulum. [file peerj-10-13511-s005.jpg]

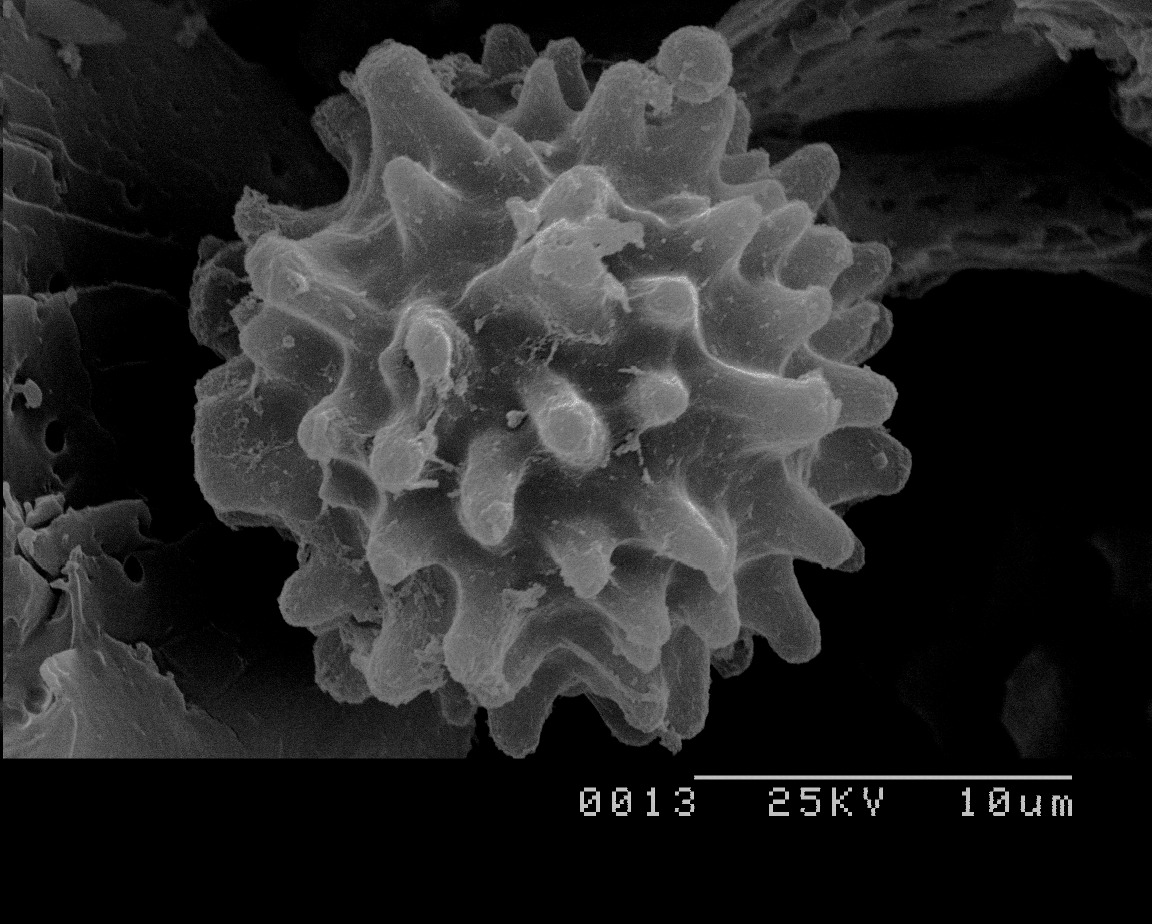

Supplement: Supplemental Information 6 — scanning electron micrography, show that ornamentation of blunt spines connected in an irregular alveolate reticulum, 1–4 μm high, mostly have a de Bary bubble and are uniguttulate, walls 1.5–2 μm thick. [file peerj-10-13511-s006.jpg]
